# Comment on "Oestrogen-induced angiogenesis and implantation contribute to the development of parasitic myomas after laparoscopic morcellation"

**DOI:** 10.1186/s12958-017-0268-z

**Published:** 2017-07-20

**Authors:** Ospan A. Mynbaev, Antonio Malvasi, Sergei S. Simakov, Andrea Tinelli

**Affiliations:** 1 Division of Molecular Technologies, Research Institute of Translational Medicine, N.I.Pirogov Russian National Research Medical University, Ostrovitianova str. 1, Moscow, 117997 Russia; 2 Institute of Numerical Mathematics, RAS, Moscow, Russia; 3 Department of Obstetrics and Gynecology, Santa Maria Hospital. G.V.M. Care and Research, Bari, Italy; 4 Division of Experimental Endoscopic Surgery, Imaging, Technology and Minimally Invasive Therapy, Department of Obstetrics and Gynecology, Vito Fazzi Hospital, Piazza Muratore, Lecce, Italy

**Keywords:** Leiomyomas, Leiomyosarcoma, Myomectomy, Power morcellators, Laparoscopic morcellation, Parasitic myomas

## Abstract

**Background:**

The cause of contamination and dissemination of leiomyoma tissue particles and cells in the peritoneal cavity during myomectomy is a challenging issue for both clinicians and researchers. Therefore, the article by Huang et al. recently published in your journal is the subject of this letter.

**Main body:**

We comment on the role of laparoscopic condition in xenograft implantation and also highlighted the shortcomings of this study. The surgical technique of intramural fibroid enucleation, cell spillage during morcellation and postsurgical hormonal impact on the development of parasitic myomas become evident, while the contribution of CO2 insufflation, the fibroid’s nature, mutations and pseudocapsule impacts on angiogenesis are not clear. In addition, an exploration of the exact origin of implanted fragments harvested from the fibroid tissue and their nature might play a significant role in the implantation and the angiogenesis induction ability of xenografts.

**Conclusion:**

Taking into account the current literature in the scope of this study, we suggest that the factors involved in development of parasitic myomas can be classified as confirmed and doubtful contributions.

## Main text

Recently, Huang et al. published an article entitled “Oestrogen-induced angiogenesis and implantation contribute to the development of parasitic myomas after laparoscopic morcellation” [1]. This study, together with other publications, addressed to causative signaling pathways of postlaparoscopic survival, growth and invasion of neoplastic cells and tissues, has an important value, since several technical laparoscopic factors, such as morcellation and CO2 pneumoperitoneum, were associated with abdominal and/or port site cancer metastasis, including leiomyosarcomas and parasitic myomas. Currently, there are crucial changes in surgical practice with the use of power morcellators for laparoscopic hysterectomies and myomectomy according to the FDA warning statement [2].

The authors suggested the laparoscopically-induced model of parasitic myomas by laparotomic fibroid tissue implantation into the peritoneal cavity in SCID mice [1]. Triggering or/and inhibiting impact of estrogen and other medications on survival, proliferation, implantation and angiogenesis induction in xenografts was demonstrated by using hormonal manipulations in either intact or ovariectomized SCID mice.

Congratulations can be expressed to the authors for such a perfectly designed study, but, while reading this article, we think it is important to know what the impact of the laparoscopic condition for xenograft implantation was, since the hormonal treatment is the main factor.

The suggested term “Laparoscopically-induced parasitic myomas” may be evidence based if the authors could demonstrate, that xenograft implantation was associated with CO2 insufflation comparing it to the relevant controls. Small fragments of fibroid tissue were implanted by laparotomic incision before the CO2 insufflation for 10 min [1]. This is not sufficient to be a causative factor for xenograft implantation and angiogenesis induction. Moreover, an impact of CO2 insufflation itself on tumor cell implantation is under debate [3]. Therefore, it seems to be more suitable to call this model as “Morcellation-induced parasitic myomas”.

Unfortunately, the authors did not describe the process of attachment of fibroid fragments to the peritoneal tissue, which could have shed light on the underlying mechanisms of the local tissue environment changes and possible involvement of fibroblasts and other cells in the xenograft implantation. It is well known that post surgical scars are preferable locations of the port-site cancer metastasis on the abdominal wall. Freely floating cells or tissue fragments in the abdominal cavity can easily attach to the fibrin deposits, and the wound healing process becomes an optimal environment for their growth and implantation.

In addition, the nature of fibroid tissue and mutations were not distinguished, although the authors mentioned these factors as the limitations of their study [1]. Thus, it is difficult to judge which part of the fibroid tissue was harvested for xenograft samples and which type of fibroid fragments was used for implantation. Taking into account the role of well known mutations and other genetic, immune and hormonal contributing mechanisms of leiomyoma cell growth induction and transformation into fibroid tissue, the findings of the above mentioned factors (fibroid type and location of harvested tissue for xenograft samples) could increase the value of this study as following reasons:

1. The actively proliferating cells are located mostly in the peripheral areas of fibroids;
2. More extracellular matrix and fewer cells are presented in the central part of fibroids;
3. The smaller fibroids contain more active cells as compared to larger ones [4]. Moreover, in vivo experiments in this study were performed by using fibroids from different patients, subsequently with heterogenous xenograft tissue in different series [1].

It is evident, that even fibroids from the same uterus can have different mutations and diverse features depending on their locations (subserosal, intramural and submucosal) and size. There is one more factor, the fibroid pseudocapsule [5] which consists of the fibro-Neuro-vascular network with the possible impact on initiating angiogenesis and vascularization in the fibroids [6, 7].

Fibroids are usually enucleated from their pseudocapsule during myomectomy independently from both open or laparoscopic approaches of surgery. Morcelation is used during laparoscopic myomectomy to evacuate large fibroids from the abdominal cavity. Dissemination of tissue particles and leiomyoma cells by spilling them into the peritoneal cavity during enucleation of intramural fibroids was suggested as the cause of parasitic myomas after open myomectomy [8, 9]. Laparoscopic morcellation is proposed as the cause of the increased incidence of parasitic myomas [10]. In addition, the recent study demonstrates an overall risk of leiomyosarcomas (12.9 per 10,000) in laparoscopic-assisted supracervical hysterectomy and myomectomy for the patients younger than 49 [11].

It can be regarded that the angiogenesis induction capacity of xenografts can be simulated and predicted by means of in silico technology [12]. This can be realized by the combination of angiogenesis, vascularisation and tumor development models. Fortunately, most of the important input data for mathematical modeling, such as immunohistochemistry data of estrogen receptor α, progesterone receptor, vimentin, vascular endothelial growth factor, microvessel density and Ki-67 index were well presented in this study.

**Fig. 1:**
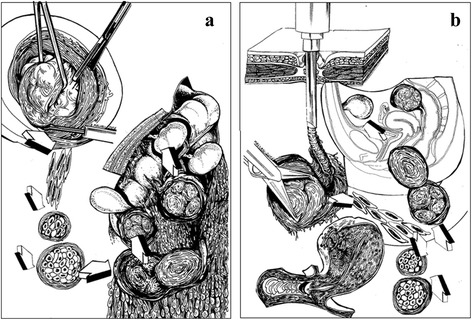
Classification of causative factors and their value in parasitic myomas development after myomectomy. **a** Leiomyoma cells and tissue fragments can be spilled in the abdominal cavity during enucleation of fibroids from their pseudocapsule with parasitic leiomyoma implantation in the wound scar tissue after open myomectomy. **b** Morcellation is an additional and more powerful factor of spilling and dissemination of leiomyoma cells and tissue fragments into the abdominal cavity with typical location of parasitic leiomyoma in port-sites

It is proposed that the factors involved in development of parasitic myomas can be classified as contributions which are confirmed or doubtful (Fig. 1).

The causative effect of the accuracy of surgical technique during enucleation of intramural fibroids, cell spillage by morcellation during fibroid evacuation and postsurgical hormonal impact is evident. However, the contribution of CO2 insufflation, the fibroid’s nature, mutations and pseudocapsule impacts on angiogenesis seems to be questionable and to be evaluated in the future.

All these factors can play a role in accordance with the seed and soil theory [13]. Thus, implanted fibroid tissue nature, mutations of leiomyoma cells and pseudocapsule potency of angiogenesis induction are likely to be important as seed promoting factors, whereas the hormonal treatment and CO2 insufflation [14] can serve as soil preparing factors in this multifactorial process.

In conclusion, this study increases the knowledge concerning the impact of different hormones and morcelation on intraperitoneal leiomyoma xenograft survival and implantation, as well as angiogenesis induction. However, it did not show the effect of CO2 insufflation, the nature of fibroids and mutations of myometrial cells upon these events. Basing on the current literature in the scope of this study, we suggest that the exact origin of implanted fragments harvested from the fibroid tissue and its nature may play a significant role in the implantation and the angiogenesis induction ability of xenografts, in addition to hormonal treatment impact as well. Even during conventional open myomectomy it is because leiomyoma cells can often be found in the abdominal cavity due to tissue spillage from fibroids.

## Abbreviations

CO2: Carbon dioxide
Ki-67: Marker of proliferation
